# Fine-Scale Population Genetic Structure and Parapatric Cryptic Species of Kuruma Shrimp (*Marsupenaeus japonicus*), Along the Northwestern Pacific Coast of China

**DOI:** 10.3389/fgene.2020.00118

**Published:** 2020-02-25

**Authors:** Panpan Wang, Baohua Chen, Jinbin Zheng, Wenzhi Cheng, Heqian Zhang, Jun Wang, Yongquan Su, Peng Xu, Yong Mao

**Affiliations:** ^1^ State Key Laboratory of Marine Environmental Science, College of Ocean and Earth Sciences, Xiamen University, Xiamen, China; ^2^ Fujian Key Laboratory of Genetics and Breeding of Marine Organisms, Xiamen University, Xiamen, China; ^3^ School of Marine Sciences, Ningbo University, Ningbo, China; ^4^ College of Marine Sciences, South China Agricultural University, Guangzhou, China

**Keywords:** *Marsupenaeus japonicus*, cryptic species, genotyping-by-sequencing, comparative transcriptomics, interbreeding experiments, reproductive isolation

## Abstract

The kuruma shrimp (*Marsupenaeus japonicus*) includes two cryptic species, which are distributed mostly allopatrically but co-occur in the northern South China Sea (from Huilai to Beihai). To obtain a better understanding of the fine-scale genetic structure and parapatric diversification of these two varieties in the northwestern Pacific region, we used a genotyping-by-sequencing (GBS) and comparative transcriptomics approach to establish their phylogenetic relationships. Using the GBS technique, we genotyped 28891 SNPs in 160 individuals in the Northwest Pacific. The results supported two highly diverged evolutionary lineages of kuruma shrimp (var. I and II). The ND and XM populations showed complex genetic patterns, which might be affected by the complex environment of the Taiwan Strait. In addition, the migration rates and inbreeding coefficients of XM and BH were much lower than those of the other populations, which might be related to the land-sea changes and complex ocean currents in the Taiwan Strait and Qiongzhou Strait. Based on the synonymous substitution rates (*ds*) of 2,491 candidate orthologs, we estimated that the divergence time between the two varieties was 0.26~0.69 Mya. Choice and no-choice interbreeding experiments provided support for the biological species concept, by showing the existence of reproductive isolation or incompatibility. In view of these differences between the two *Marsupenaeus* species, we believe that it is essential and urgent to establish a genetic database for each and reevaluate their ecological suitable conditions in order to improve species-specific culturing techniques. Moreover, this research can serve as a case study for future research on speciation and hybridization.

## Introduction

The northwestern (NW) Pacific marginal seas comprise approximately 75% of the world’s marginal seas (Tamaki, 1991). Due to climatic fluctuations associated with Pleistocene glaciation-interglaciation, the shoreline and configuration of these marginal seas changed dramatically (Wang, 1999; Ni et al., 2014). Repeating drastic changes and complex ocean currents together shape the abundant geographic variation and ecological adaptation of marine animals. Comparative molecular phylogeography fuses population genetics and phylogenetics, and combines molecular genetics, statistics, ecology and biogeography (Avise, 2009). Population diversity and adaptive divergence are critical for predicting the evolutionary potential, of both populations and species (Therkildsen et al., 2013). Due to ubiquitous cryptic and sibling species, the geographical distribution of marine biodiversity is often underestimated (Bickford et al., 2007; Taylor et al., 2017; Jorde et al., 2018). Cheng et al. revealed the allopatric speciation and recent hybridization of two *Oratosquilla oratoria* cryptic species using the mtDNA COI and nrDNA ITS genes (Cheng and Sha, 2017). Two phylogeographic lineages of *Bostrychus sinensis* underwent secondary contact and hybridization in the East China Sea (Qiu et al., 2016; Ding et al., 2018). Shen et al. documented no gene flow among three cryptic species of *Mugil cephalus* in the NW Pacific, indicating complete speciation (Shen et al., 2011).

The kuruma shrimp (*Marsupenaeus japonicus*), a commercially important crustacean, is widely distributed in the East and South China Sea, the region off Australia and the western Indian Ocean (Tsoi et al., 2014). Traditionally, *M. japonicus* was regarded as the only species of *Marsupenaeus* (Tirmizi, 1971; Lavery et al., 2004). A study by Tsoi et al. showed that kuruma shrimp had two morphologically similar varieties, namely, Forms I and II, which were characterized by their carapace banding patterns (Tsoi et al., 2005). Form I was confined to the East China Sea and northern South China Sea, while Form II was distributed in the South China Sea, Australia, and Southeast Asia seas. Tsoi et al. indicated that the two forms were overlapped in the northern South China Sea and Taiwan by sampling mixed individuals from Hong Kong and Taiwan (Tsoi et al., 2007). However, the certain sympatric range had not been showed because of vague samples. In addition, previous laboratory results indicated that the sympatric areas were limited to the Huilai-adjacent sea area (He et al., 2012). It is important to determine the areas of sympatry and understand the ecological differences of sympatric cryptic species, which are valuable systems for exploring gene exchange, introgressive hybridization, and species differentiation (Michel et al., 2010; Addison and Kim, 2018).

Several previous studies on the population genetic structure of kuruma shrimp were based on mt-DNA and a few simple sequence repeats (SSR) markers. Mitochondrial genes are maternally inherited, which makes them inappropriate for the detection of hybridization (Ballard and Whitlock, 2004; Mccauley et al., 2005; Ravago-Gotanco et al., 2018). These population genetic studies provided substantial evidence for the existence of two phylogeographic lineages. However, a handful of genetic markers is not appropriate for inferring fine-scale population structure and genetic differentiation, especially in cryptic species (Struck et al., 2018; Pedraza-Marrón et al., 2019). Recent rapid developments in next-generation sequencing (NGS) have provided many extraordinary tools with which to study population divergence (Davey et al., 2011; Xu et al., 2016; Clucas et al., 2018; Friedline et al., 2019; Rincon-Sandoval et al., 2019; Vendrami et al., 2019). The genotyping-by-sequencing (GBS) technique can rapidly generate considerable numbers of genome-wide genetic markers, which has revolutionized the field of ecological and evolutionary genomics (Elshire et al., 2012; Andrews et al., 2016; Hume et al., 2018). Using the GBS technique, increasing numbers of studies have revealed fine-scale population genetic structure (Near et al., 2018; Palaiokostas et al., 2018; Robledo et al., 2018; Zhao et al., 2018). Most cryptic species have been distinguished by DNA-barcoding and other genetic methods, while few have been verified to be reproductively isolated or incompatible using interbreeding experiments (Kress et al., 2015; Lehnert et al., 2016; Paterson et al., 2016). As speciation remains controversial, different scholars have used different definitions and criteria, and it is difficult to accurately define cryptic or sibling species based on only one type of data (Bolnick and Fitzpatrick, 2007; Fitzpatrick et al., 2008; Mallet et al., 2009; Fišer et al., 2018; Struck et al., 2018; Pedraza-Marrón et al., 2019).

In this study, we investigated the areas of sympatry and whether there is hybridization among the wild populations by sampling from a narrow sympatric zone. Furthermore, we combined orthologous genes with genotyping-by sequencing (GBS) to establish the fine-scale population structure and phylogenetic relationships of kuruma shrimp in the NW Pacific region. To verify the existence of reproductive incompatibility, we implemented choice and no-choice interbreeding experiments in purse seines. This study provides comprehensive insight into the two *Marsupenaeus* species, which will facilitate further studies on the molecular mechanisms underlying genetic differentiation.

## Materials and Methods

### Sample Collection

**Figure 1:**
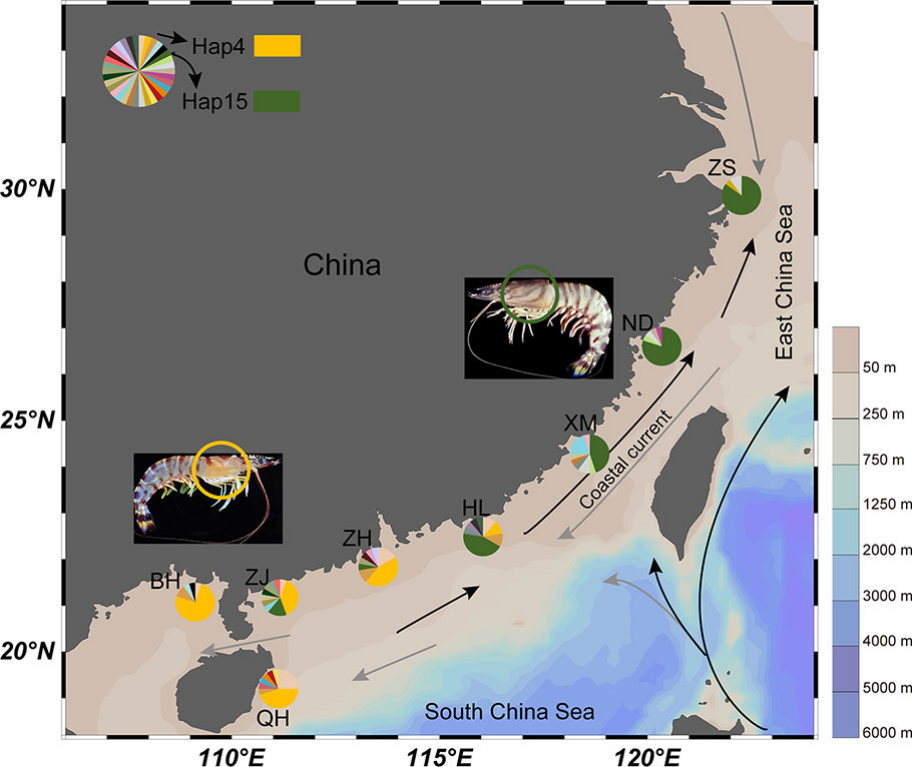
Map showing geographic locations of kuruma shrimp. The lines represent the ocean currents and arrows indicate direction (black represent summer and gray represent winter). The pie charts represent haplotype composition. ZS, Zhoushan; ND, Ningde; XM, Xiamen; HL, Huilai; ZH, Zhuhai; ZJ, Zhanjiang; BH, Beihai, QH, Qionghai.

Kuruma shrimp samples were collected from eight locations along the coast of China from September 2016 to May 2018 (**Figure 1**). The samples were collected directly from local fishermen. All samples were collected in accordance with the national legislation of the countries concerned. The morphological and sexual characters were identified and recorded, and part of the abdominal muscle was preserved in 100% ethanol for subsequent DNA extraction. Genomic DNA from 160 individuals was isolated from muscle tissues using a DNeasy Blood and Tissue Kit (Qiagen, Germany) following the manufacturer’s instructions. DNA integrity and purity were visualized by 1% agarose gel electrophoresis. DNA purity and concentration were measured using a NanoPhotometer^®^ (Implen, CA, USA) and Qubit fluorometer (Life Technologies, CA, USA), respectively. Only nondegraded samples with an OD260/280 from 1.7 to 1.9 were subjected to genotyping-by-sequencing. The hepatopancreas tissues of ten HL1 individuals (var. I, mean weight: 12.67 g) and ten HL2 individuals (var. II, mean weight: 11.36 g) individuals from the Huilai population were rinsed separately with RNase-free water for RNA extraction.

### Estimation of Substitution Rates and Phylogenetic Analysis Based on Transcriptomes

Comparative transcriptome libraries were sequenced on an Illumina HiSeq X-Ten platform with 150-bp paired-end reads at Novogene Bioinformatics Technology Co., Ltd. (Beijing, China). Orthologous genes were screened by OrthoMCL (Li et al., 2003). Subsequently, synonymous substitution rates (*d*_S_) were calculated using PAML (Phylogenetic analysis by maximum likelihood). In this study, we screened a single copy nuclear genes (SCNGs), which was annotated as *caspase* (EF079670.1), and the *caspase* gene has high sequence diversity (Bin et al., 2011). DnaSP v6 (Rozas et al., 2017) and Network software (Bandelt et al., 1999) were used to analyze the haplotype data.

### Genotyping-by Sequencing (GBS) Library Construction and Sequencing

Libraries were constructed following the GBS protocol described by Elshire et al. (2012), with minor modifications. Briefly, total gDNA from each sample was completely digested with the restriction enzyme HaeIII to obtain suitable markers. The P1 and P2 adapters with barcodes were ligated to the sticky ends of the digested fragments by adding T4 ligase (New England Biolabs, USA). Twenty samples with equal molarities from each population were pooled together. The restriction fragments with ligated adapters were amplified to construct sequencing libraries. To ensure quality, the libraries were quantified using a Qubit 2.0, and the concentration was diluted to 1 ng/μl. All libraries were sequenced on the Illumina HiSeq 4000 platform with 150-bp paired-end reads at Novogene Bioinformatics Technology Co.Ltd (Beijing, China).

### Data Processing and SNP Genotyping

The original data obtained by high-throughput sequencing were transformed to raw reads by base calling. Clean data were obtained by removing reads containing adapters, ploy-N sequences and low-quality bases from the raw data. Then, the enzyme catch ratio was calculated to evaluate the efficiency of enzyme digestion. The high-quality clean reads were mapped to the reference sample (XM08) using the Burrows-Wheeler Aligner (BWA) with the command ‘mem-t4-k32-M’ (Li and Durbin, 2009). The SAMtools package (Li et al., 2009) was used to call candidate SNP markers from the alignment results for subsequent analysis. High-quality SNP sites were obtained by screening with the parameter dp2, miss0.9 and minimum allele frequency (MAF) > 0.01. Subsequently, we tested for Hardy-Weinberg equilibrium (HWE) and linkage disequilibrium between each pair of loci by the program PLINK v.1.9 (Purcell et al., 2007).

### Population Genetic Polymorphism

Following SNP detection, vcf files were converted to other formats by PGDSpider for downstream analysis (Lischer and Excoffier, 2012). The observed heterozygosity (*H_o_*) analysis, expected heterozygosity (*H_E_*) analysis and Analysis of Molecular Variance (AMOVA) were performed in the Arlequin v3.5 (Excoffier and Lischer, 2010). VCFtools (Danecek et al., 2011) was used to calculate the within-population genetic differentiation index (*Fst*) and nucleotide diversity (π). Isolation-by-distance mantel tests were performed to examine correlations between genetic distance and geographic distance matrices by IBD (Isolation By Distance) software (Bohonak, 2002). Populations v1.2 (http://www.bioinformatics.org/project/?group_id=84) was used to produce the pairwise population matrix of Nei’s standard genetic distances (*Dst*). To estimate contemporary migration rates, we used the Bayesian algorithm implemented in BayesAss software (v 3.0.4) (Wilson and Rannala, 2003).

### Population Structure Analysis

Phylogenetic analyses were performed for all high-quality SNP sites, and the individual SNPs were used to calculate the distance among populations. The distance matrix, calculated using TreeBeST v1.9 software (Vilella et al., 2009), was used to construct a phylogenetic tree with the neighbor-joining method with 1,000 bootstrap values. Phylogenetic trees were constructed for all individuals, which visually display the evolutionary relationship between different populations and varieties. To illustrate the genetic relationship of different populations, principal component analysis (PCA) of 160 individuals was conducted based on SNPs among individual genomes using the program GCTA v1.25 (http://cnsgenomics.com/software/gcta/#PCA) and visualized by R language. The population genetic structure and ancestry of each sample were analyzed by the program FRAPPE v1.1 (Hua et al., 2005) with 10^5^ burn-in iterations and 5*10^5^ Markov chain Monte Carlo iterations. We further used Admixture (Alexander et al., 2009) to determine the most likely number of genetic clusters and validate the population structure constructed by FRAPPE.

### Detecting of Wild Hybrid Individuals and Interbreeding Experiments

The two varieties have only subtle morphological differences in carapace color banding patterns. We screened the cytochrome b (*Cytb*) mitochondrial gene and sodium-potassium ATPase alpha-subunit (*NaK*) gene to detect hybridization. Mitochondrial genes are maternally inherited, and nuclear genes contain genetic information from both parents. It was a rapid and accurate method for interspecific hybrids identification by combining *Cytb*, *NaK* and available sequences of NCBI (National Center for Biotechnology Information). About 600 wild individuals from sympatric areas Huilai, Zhuhai and Zhanjiang were detected by amplifying the *Cytb* and *NaK* gene sequences. Meanwhile, we performed the choice and no-choice interbreeding experiments for detecting the reproductive isolation between two varieties. Two hundred healthy individuals, approximately 100 each of var. I (mean weight, 73.83 ± 20.37 g) and var. II (mean weight, 65.34 ± 12.52 g), were transported from Huilai to an aquaculture farm in Dongshan (Fujian) and acclimated for four weeks (23°C, 28 salinity) until the primary molt was complete. The rest of the healthy individuals (59% for var. I and 79% for var. II) were used for interbreeding experiments. The seawater was renewed every 2 d, and the shrimps were fed twice daily with oysters or squid. We assessed the mating condition of female individuals by checking spermatophores every 3 d. Once mating was completed, the female individuals were moved to a prepared 5 m^3^ circular barrel. On the second day, the eyestalk on one side was cut off disinfected by 0.5% povidone iodine solution. Thereafter, the females were fed nereids to stimulate ovarian development.

## Results

### Estimation of Differentiation Time and Phylogenetic Analysis

**Figure 2:**
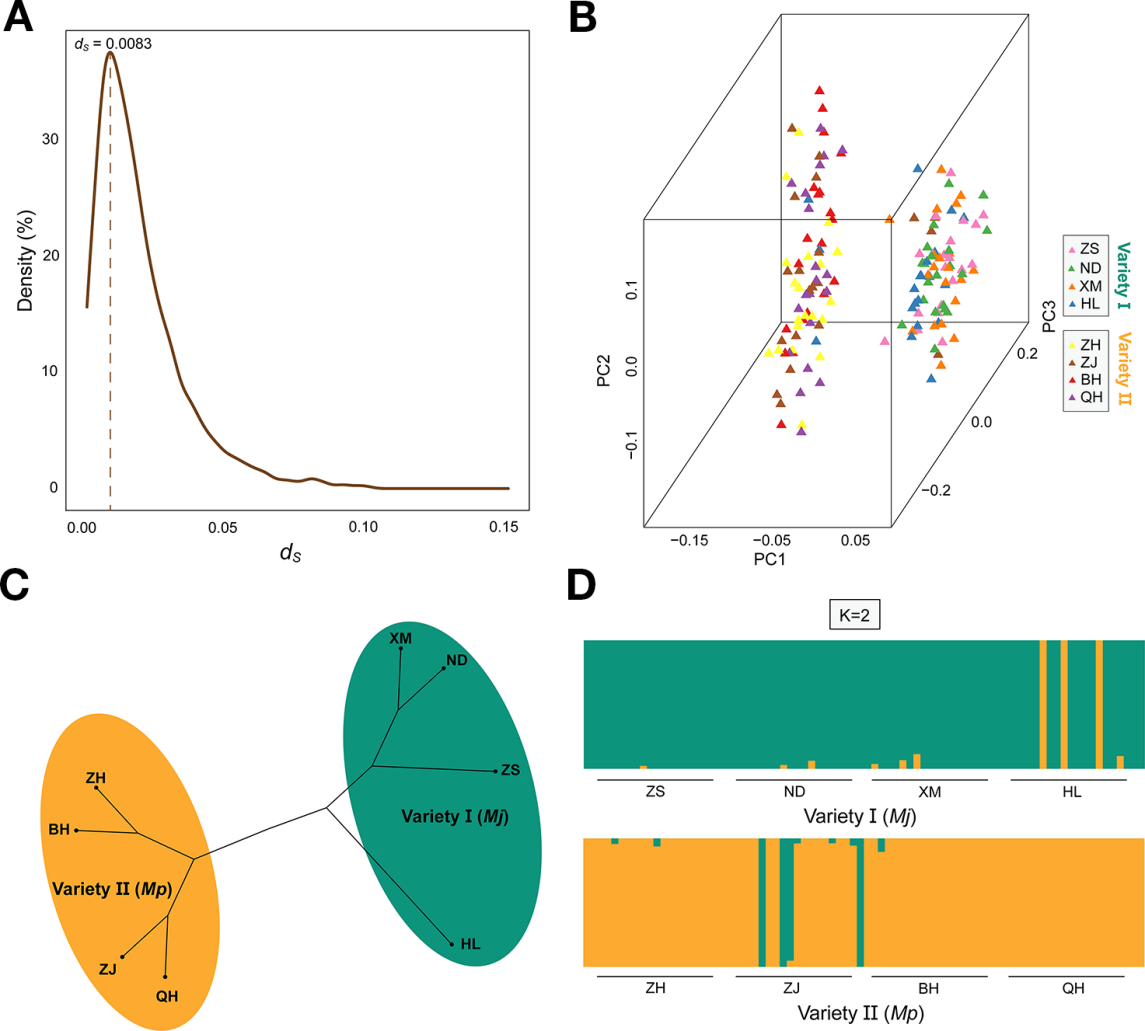
The ds value density distribution and the peak was signed **(A)**. Principle component analysis (PCA) **(B)**. The UPGMA clustering tree based on pairwise distances for eight populations **(C)**. Bayesian plot of ancestral fractions from the admixture analysis. Each vertical column represents one individual **(D)**.

We sequenced and compared the hepatopancreas transcriptomes of HL1 (var. I) and HL2 (var. II) (**Table S1**), and identified 5036 pairs of putative orthologs. The detailed introduction could be found in Wang et al. (Wang et al., 2019). Using the PAML CodeML package, we calculated the *d*_N_ */d*_S_ (ω) ratios of 2491 pairs of orthologous genes (**Table S2**), and the peak in the *d*_S_ value distribution was at 0.0083 (**Figure 2A**). The average *d*_S_ rate for the nuclear genes is approximately 2×10^-9^ ~ 16×10^-9^ substitutions per synonymous site per year for higher animals and *Drosophila* (Li et al., 1987; Sharp and Li, 1989). According to the formula: T = K/2r (Graur and Li, 2000), we estimated the divergence time between variety I and variety II to be approximately 0.26 ~ 0.69 Mya. The *caspase* gene (ω value = 1.22) was amplified from 160 genomic DNA samples. There were 57 unique haplotypes, with high diversity (0.891). Network analysis identified two well-supported clades consisting of the expected lineages of variety I (ZS, ND, XM, and HL) and variety II (ZH, ZJ, BH, and QH) (**Figure 1**). The two varieties each had a dominant haplotype (Hap15 and Hap4) and shared six haplotypes.

### GBS Mapping and SNP Genotyping

GBS sequencing of 160 samples produced 112.45 Gb of raw reads, with an average of 0.7Gb per individual. After quality filtering, a total of 112.44 Gb of clean reads was retained, which represented an average effective rate of 99% (**Table S3**). The sequencing results showed that the average Q20 values, Q30 values and GC content were 93.66%, 86.05% and 38.7%, respectively (**Table S4**). Due to the lack of a reference genome, clean reads from the high-coverage-depth sample were assembled as references for downstream analyses. After screening by SAMTOOLS, a total of 28,891 SNPs for all populations, 24,861 SNPs for var. I populations (ZS, ND, XM and HL), and 15,904 SNPs for var. II populations (ZH, ZJ, BH and QH) were obtained. The number of usable SNPs among the eight populations ranged from 2,751 loci in the ZH population to 7,669 loci in the XM population. For all eight populations, 17,937 SNPs were putative transitions (Ts), and 10,954 SNPs were putative transversions (Tv) with a Ts : Tv ratio of 1.64. In addition, we analyzed the pairwise SNP markers between all populations, and the results showed that the number of SNPs shared between varieties was significantly lower than that shared within varieties (**Figure S1**). In addition, we compared the special samples of HL and ZJ with the others in the same population and sex differences in var. I and var. II.

### Genome-Wide Analysis of Genetic Diversity and Differentiation

The genetic diversity based on all SNP loci was computed in the eight populations (*H_E_*, 0.2447~0.28842; *H_O_*, 0.30286~0.38663; π, 0.0018~0.0032) (**Table S5**). The mean *H_O_* of 0.357 was significantly higher than the mean *H_E_* of 0.269 (*p* < 0.001), which suggests heterozygote excess among samples. The AMOVA results showed that 92.4% of the genetic variation was within populations and only 7.6% occurred among populations (**Table S6**). Pairwise *Fst* values showed that the var. I and II populations were strongly and significantly genetically differentiated, with *Fst* values ranging from 0.0964 (ZJ vs HL) to 0.3378 (BH vs ND) (**Figure S2A**). The mean *Fst* values within varieties were 0.0064 for var. I and 0.0067 for var. II, which indicated a little genetic differentiation. Based on a Mantel test, the correlation between genetic similarity and log (geographic distance) was statistically significant for all eight populations (R^2^ = 0.534) (**Figure S2B**). However, the correlation for both var. I (R^2^ = 0.002) and var. II (R^2^ = 0.229) was insignificant. The calculation results of contemporary migration rates showed that recent migration rates from var. I to var. II and vice versa were very low (**Figure S3**), suggesting that gene flow between var. I and II was substantially blocked. Pairwise migration rates showed that the migration from ZS, ND, and HL to XM was much higher than that in the other direction. Similarly, the rates from ZH, ZJ, and QH to BH were much higher than those were in the other direction. The mean posterior estimates of inbreeding coefficients (Ic) showed that the values of XM and BH were 0.003 and 0.0009, which were much lower than the values of the other populations.

### Population Genetic Structure Analysis

**Figure 3:**
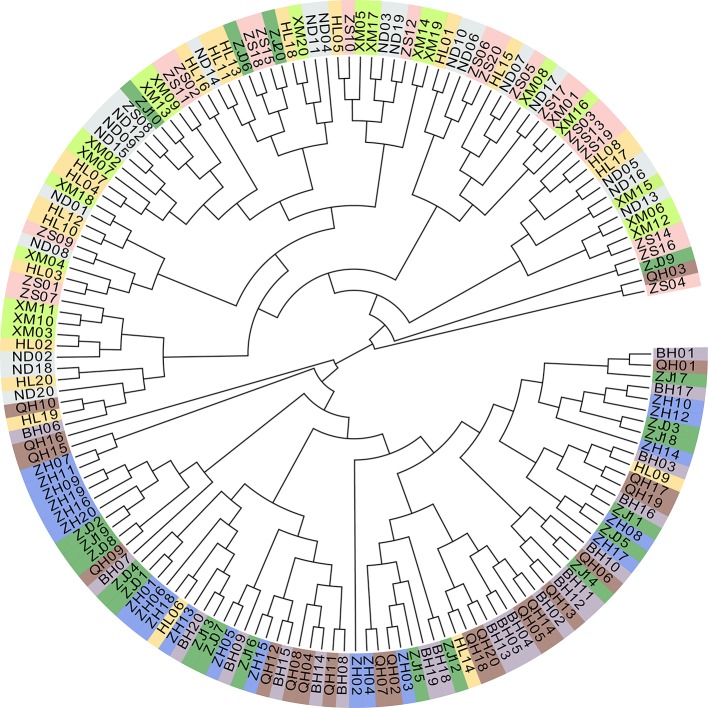
Phylogenetic tree for all individuals.

Principle component analysis demonstrated that the ZS, ND, XM, HL samples were clustered with var. I on the left and ZH, ZJ, BH, QH samples were clustered with var. II on the right (**Figure 2B**). Additionally, the special individuals from the HL (6/9/14) and ZJ (6/9/10/20) populations were clustered with var. II and I, respectively, which was in accordance with their morphological characteristics. The phylogenetic analysis of all eight populations based on genetic distance showed that var. I (ZS, ND, XM, HL) and var. II (ZH, ZJ, BH, QH) formed two distinct clusters with little genetic variation within each cluster (**Figure 2C**). To some extent, the phylogenetic tree displayed a similar topological pattern reflecting the geographic distribution of all populations. The cluster analysis implemented in FRAPPE revealed the optimal K value was 2 and the eight populations were split into two groups, namely, var. I and var. II (**Figure 2D**). It was interesting to see that the special samples of HL6/9/14 and ZJ 6/9/10/20 retained pure genetic information. Based on distance matrix, the phylogenetic trees (**Figure 3**) were constructed for all individuals, which formed two distinct clusters similar to the result of structure.

### Identification of Wild Hybrid Individuals

**Figure 4:**
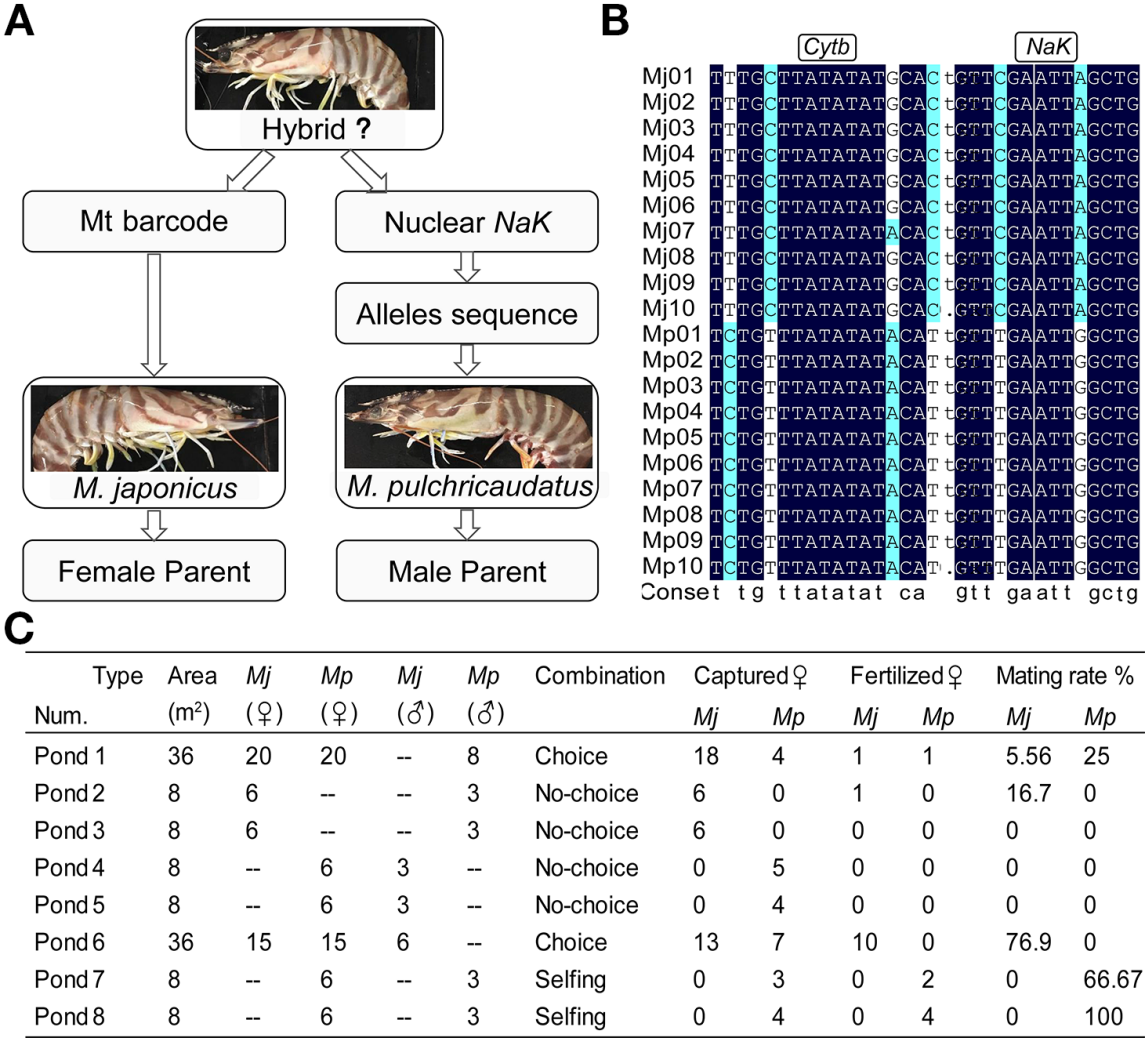
Establishment of hybrid evaluation system **(A**, **B)**. Choice and no-choice interbreeding experiments **(C)**.

The two morphologically similar varieties have only subtle differences in carapace color banding patterns. The Zhoushan (ZS, var. I) and Qionghai (QH, var. II) populations have pure genetic backgrounds. Firstly, we amplified the *Cytb* and *NaK* sequences of about one hundred ZS individuals and one hundred QH individuals, and found stable heterozygous loci (**Figures 4A, B**). Subsequently, we amplified the *Cytb* and *NaK* genes of about 600 wild individuals from sympatric areas. Phenotypes (carapace pattern) of all individuals were consistent with genotypes (**Figures S4** and **S5**). Amplicons were unimodal in the detected base loci. These results indicated that there was no natural hybridization in wild populations.

### Choice and No-Choice Interbreeding Experiments

A total of 138 individuals were divided into eight breeding-pair treatments in purse seines (**Figure 4C**). After 3 months of culture, the mating condition of individuals in each purse seine was recorded, as shown in **Figure 4C**. Overall, the selfing rates were higher than the hybridization rates, with the selfing rates of var. *I* higher than those of var. II. In the beginning, there were 20 individuals per variety in the purse seine one. However, we captured only four individuals of var. II and were unsure of the conditions they had experienced. By comparing the different treatments, we found that the attraction within varieties was significantly higher than that between varieties. Our primary focus was cross-combinations, most of which failed. However, fortunately, we obtained two hybrid individuals from purse seines 1 and 2, both of which were var. I females. The two individuals were numbered N0101 and N0201 and then moved to a five m^3^ circular barrel. It was strange that the two individuals did not complete spawning and this phase lasted 6 d, ending up shedding the spermatophore.

## Discussion

### Population Distribution Characteristics

In the present study, we investigated and collected kuruma shrimp samples along the coast of China. We found that the sympatric areas range from Huilai (Guangdong) to Beihai (Guangxi) with a tendency change to some extent. In previous studies by Tsoi et al. (2014), *M. japonicus* (var. I) was found to be confined to the East China Sea (including Japan) and the northern South China Sea. Var. II (called *M. pulchricaudatus*) was widely distributed in the South China Sea, Australia, the Red Sea, the Mediterranean and the western Indian Ocean. However, the authors did not explicitly identify the sympatric areas. Tsoi et al. (2007) mentioned that two and six individuals from Hong Kong and Taiwan were identified as variety II, respectively, and the others were variety I. However, the author stated that the origin of these samples was unknown. Our previous investigations showed that the sympatric area was limited to Huilai (Guangdong) (Zeng et al., 2010; He et al., 2012). The kuruma shrimp, having a nocturnal habit, mainly inhabits 10~40 m depths. As a subtropical species, kuruma shrimp have broad temperature adaptability, and the appropriate temperature range is 24~29°C. However, kuruma shrimp will stop eating at 8~10°C and die below 5°C. The threshold temperature of embryo development is 20~32°C. In the Yellow Sea, the temperature gradually decreases form December to February. Even in summer, the cold water area (below 10°C), which is influenced by the YSCWM (Yellow Sea Cold Water Mass), covers a third of the Yellow Sea’s total area (Li et al., 2015). Our previous research revealed that the thermotolerance of var. II was stronger than that of var. I by comparing CTMax values and acclimation response ratio (ARR) values (Song et al., 2014). Teske et al. indicated that temperature-mediated diversifying selection may be an important early-stage factor in the evolution of marine biodiversity (Teske et al., 2019). In addition, the Yangtze River discharge and diluted water of the Zhujiang (Pearl) River have a significant effect on sea-surface salinity (SSS) (Delcroix and Murtugudde, 2002; Zhou et al., 2012). The larvae of *M. japonicus* were hyper-osmoconformers and has a weak salinity tolerance (Dalla Via, 1986; Charmantier et al., 1988). Thus, the natural environment in the north of the Yangtze estuary is not suitable for kuruma shrimp survival. In summary, the temperature and salinity were major limiting factors.

### Genomic Population Structure

Our study reported the generation of genome-wide SNPs for kuruma shrimp using the GBS-seq approach. This is the first study to construct the fine-scale population structure of kuruma shrimp along the Chinese coast. For technical reasons, previous studies relied on mitochondrial genes and several microsatellite markers, which cannot depict genetic structure in detail, especially for cryptic species. The average of the pairwise *Fst* values, including in sympatric areas, was 0.263 with weak gene flow, which indicated strong genetic differentiation (Wright, 1978). Our results showed that recent migration rates both from var. I to var. II and vice versa were very low. Reinforcement theory holds that reinforcement is nearly identical to later stage of speciation, which is an increase in prezygotic isolation between hybridizing populations (Howard and Gregory, 1993; Servedio and Noor, 2003; Dyer et al., 2018). Although gene exchanges does inhibit the speciation process, it is the proportion of migrants (*m*) exchanged rather than the number of migrants (*Nm*) that matters (Porter and Johnson, 2002; Panova et al., 2006). Sota et al. revealed that the diverged populations of *Parafontaria tonominea* underwent restricted dispersal and secondary contact without hybridization (Sota and Tanabe, 2009). The Mantel test showed that genetic similarity and geographic distance were significant positively correlated, which conformed to isolation as distance by Wright (1943). The fine-scale genetic structure showed that special individuals in the HL and ZJ populations had a pure genetic background. In addition, the HL6/9/14 and ZJ6/9/10 individuals were all female, and ZJ20 was male, which may indicate reproductive isolation or incompatibility between the two varieties and guide the next crossbreeding experiments. We used the mitochondrial and nuclear gene to identify suspected hybridization. This approach has been shown to be effective in abalone (You et al., 2014) and groupers (Qu et al., 2015). We did not detect any hybrids, even though some individuals from sympatric areas had carapace banding patterns different from those of var. I and II. Therefore, these special samples did not provide insights into hybridization events. Due to the lack of reference genome, we could not obtain detailed annotation information, such as sex differences and environment-related genes.

### Transcriptome Divergence Between Two *Marsupenaeus* Species

Our data provided confirmatory evidence for the obvious genetic divergence of two varieties (var. I and II), which was consistent with the findings of previous studies based on limited SSR markers and mitochondrial genes (Tsoi et al., 2007; He et al., 2012; Tsoi et al., 2014). Both within varieties and in all populations, the XM and ND populations exhibited complex genetic patterns, which might be related to the land-sea changes and complex ocean currents in the Taiwan Strait. By comparative transcriptome analysis, the divergence time between the two varieties was estimated to be 0.26~0.69 Mya, which was in the middle Pleistocene [Marine Isotope Stage 9~16, (Railsback et al., 2015)]. During the middle Pleistocene, shallow seafloors along the Southeastern coast of China including the Taiwan Strait experienced transgressive-regressive cycles (Wang, 1999; Voris, 2000; Ni et al., 2014). The divergence time obtained here was later than the time estimated based on mitochondrial genes (1.1~4.7 Mya) by Tsoi et al. (2005; 2007). Although there was no obvious spawning migration, kuruma shrimp presented regional clustering phenomenon, and migration mainly depended on the dispersal ability of planktonic larvae. During the spring and summer, adult individuals spawn in shallow water, facilitated by the China Coastal Current and Kuroshio Current that flow northeastwardly across the Taiwan Strait. Throughout the year, the ocean current in the Qiongzhou Strait essentially travels east to west, and the South China Sea water flows into Beibu Gulf through the Qiongzhou Strait (Dasen, 2006). Therefore, the BH populations are unique, which can be seen from their migration rates and inbreeding coefficients.

### Identification of Hybrids

We amplified and compared the *Cytb* and *NaK* genes of about 600 sympatric individuals and the carapace coloring patterns were in accordance with genotypes. There was no natural hybridization in wild populations based on the current sample size. In the eight breeding treatments, only two var. I females mated with var. II males. The successful selfing rate was significant higher than that of hybridization, with var. *I* exhibiting higher values than var. II. Unfortunately, the two individuals did not complete spawning, ending up shedding the spermatophore. Only a few examples of interspecies hybridization between penaeids were using spermatophore transplantation, and spawn rate, hatch rate and the survival of hybrids were lower than that of intraspecific matings (Bray et al., 1990; Benzie et al., 1995; Lin et al., 1998). Misamore et al. indicated that no spontaneous matings were observed between *penaeus setiferus* and *penaeus vannamei*, and no interspecific crosses were fertile in the artificial insemination (Misamore and Browdy, 1997).

Previous laboratory studies have shown significant differences in the morphology (L/BL and H/BL) of seminal vesicles by comparing dozens of var. I and II females at different developmental stages (III, IV, and V). Landry et al. revealed the rapid evolution of gamete recognition and sperm morphology of *Echinometra* cryptic species in the past 250,000 years (Landry et al., 2003). Sexual morphological divergence affected mating compatibility and resulted in mechanical reproductive isolation between sympatric *Parafontaria tonominea* species (Sota and Tanabe, 2009). In addition, the survival rate of var. II was lower than that of var. I, especially in the purse seine 1, which we speculate was due to several reasons, such as competition, sexual selection, environmental suitability and limited space. Among these factors, the temperature may be the major limitation and var. II individuals have poor low-temperature tolerance, which limit northward spread. Cryptic and sibling species have different habitat preferences defined by abiotic factors, such as depth, temperature, salinity and dissolved oxygen (Wellenreuther et al., 2007; Dennis and Hellberg, 2010; Niemiller et al., 2013; Gabaldón et al., 2015; Yasser et al., 2018).

## Conclusion

Overall, we conclude the occurrence of prezygotic reproductive isolation between the two varieties, which prevents natural hybridization. This isolation mechanism also explains the incomplete speciation (Kautt et al., 2016; Turissini et al., 2017; Plough et al., 2018; Raphael et al., 2019). This laboratory crossbreeding experiment and wild populations failed to obtain hybrid offspring, which indicated that reproductive isolation exists between the two varieties. Work to date suggests that the two forms exhibit wide variation in many aspects, including their phenotypes, seminal vesicles, temperature tolerance, and molecular sequences (mitochondria and nuclear genes). Therefore, we believe that the two morphologically similar varieties (I and II) are two separate species. Additionally, we support Tsoi’s nomenclature: *Marsupenaeus japonicus* (Form I) and *Marsupenaeus pulchricaudatus* (Form II) (Holthuis et al., 1993; Tsoi et al., 2014). As Tsoi et al. reported, it is essential to improve species-specific culturing techniques for these two species. In the future, we will evaluate and compare various traits between the two *marsupenaeus* species, including multiple environmental factors. In addition, a more optimized hybrid experiment and a mating behavioral study will be implemented to confirm reproductive isolation. The results of this study provided comprehensive insight into the two *marsupenaeus* species, which not only will facilitate further studies on the molecular mechanisms underlying genetic differentiation, but also can serve as a case study for future research on speciation and hybridization.

## Data Availability Statement

The datasets generated for this study can be found in NCBI BioProject PRJNA489160 and PRJNA596055.

## Ethics Statement

All experimental procedures were conducted in conformity with institutional guidelines for the care and use of laboratory animals in Xiamen University, Xiamen, China.

## Author Contributions

PW, YM, and PX conceived and designed the experiments. YM, YS, and JW obtained funds for the study. PW, WC, and HZ completed sample collection. PW, JZ, WC, and HZ designed and implemented the choice and no-choice interbreeding experiments. PW and BC conducted computational and statistical analyses. PW, BC, and YM drafted the manuscript. PX, YM, YS, and JW participated in the manuscript revision. All authors read and approved the final manuscript.

## Funding

This project was funded by the Project of China Agriculture Research System (Grant No. CARS-48), the Marine Economy Innovation and Development Project of Xiamen (Grant No. 16CZY009SF05), Special funds for Ocean and Fishery Structure Adjustment of Fujian Province in 2019 (Fujian Financial Index No. [2018]140), Science and Technology Plan Project of Ningbo (2019B10011), the Industry-University Collaboration Project of Fujian Province (2019N5001), the State Key Laboratory of Large Yellow Croaker Breeding (Fujian Fuding Seagull Fishing Food Co., Ltd) (Nos. LYC2017ZY01, LYC2017RS05), the Fundamental Research Funds for the Central Universities (Nos. 20720180123 & 20720160110).

## Conflict of Interest

The authors declare that the research was conducted in the absence of any commercial or financial relationships that could be construed as a potential conflict of interest.

## Supplementary Material

Figure S1 Venn diagram of SNP markers, between populations **(A)**, HL and ZJ **(B)**, and gender **(C)**.

Figure S2 Pairwise Fst between eight populations **(A)** and the bivariate plot of log geographic distance against Fst/(1-Fst) values **(B)**.

Figure S3 Recent migration rates and inbreeding coefficients estimation among the eight sampling locations. Ic = inbreeding coefficients.

Figure S4 Partial Cytb fragments of individuals from sympatric areas.

Figure S5 Partial NaK fragments of individuals from sympatric areas.
