# Supplementary figures and images for: Fine-Scale Population Genetic Structure and Parapatric Cryptic Species of Kuruma Shrimp (Marsupenaeus japonicus), Along the Northwestern Pacific Coast of China
Source: Front Genet. 2020 Feb 25;11:118. doi: 10.3389/fgene.2020.00118 (PMC7052491; doi:10.3389/fgene.2020.00118)

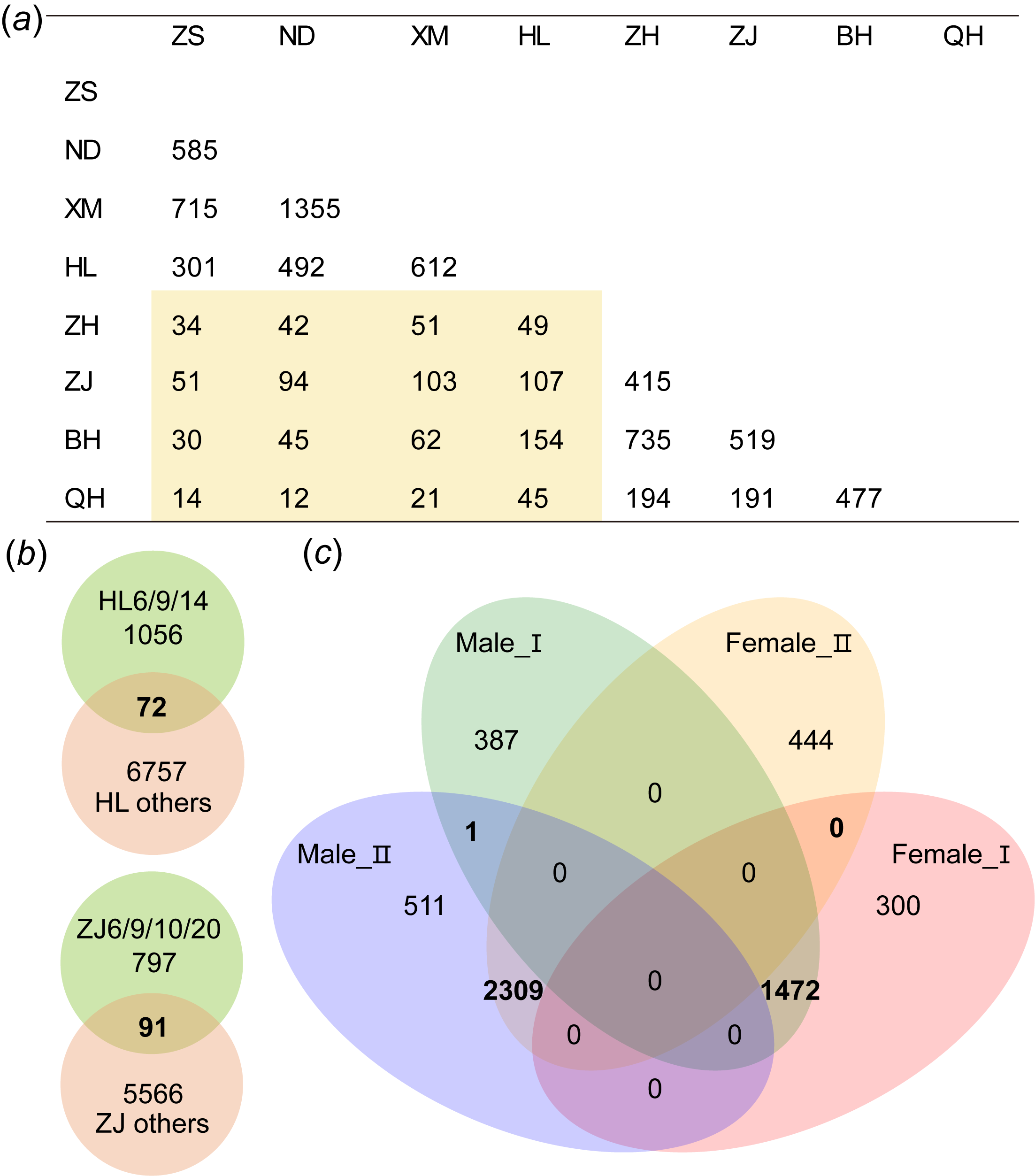

Supplement: Figure S1 — Venn diagram of SNP markers, between populations (A), HL and ZJ (B), and gender (C). [file DataSheet_1.zip › Figure S1.tif]

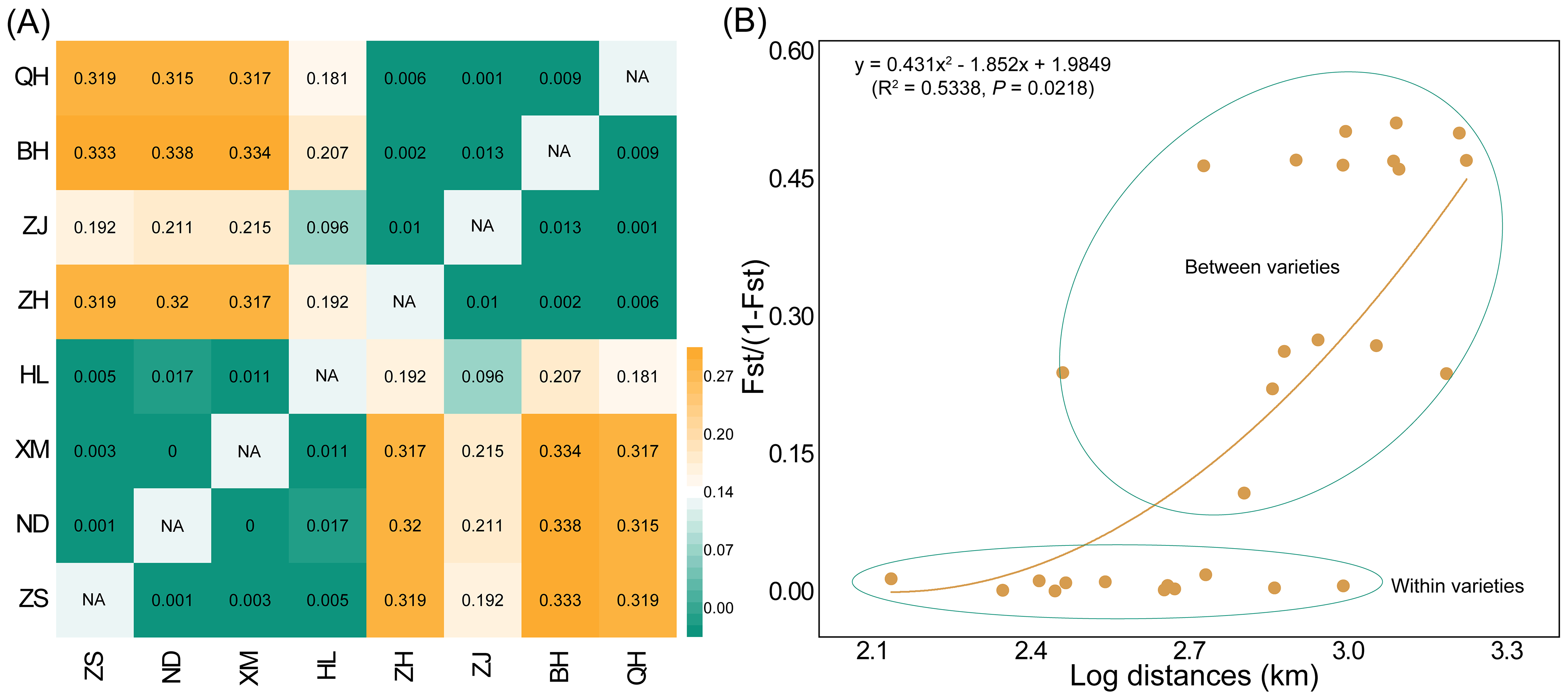

Supplement: Figure S1 — Venn diagram of SNP markers, between populations (A), HL and ZJ (B), and gender (C). [file DataSheet_1.zip › Figure S2.tif]

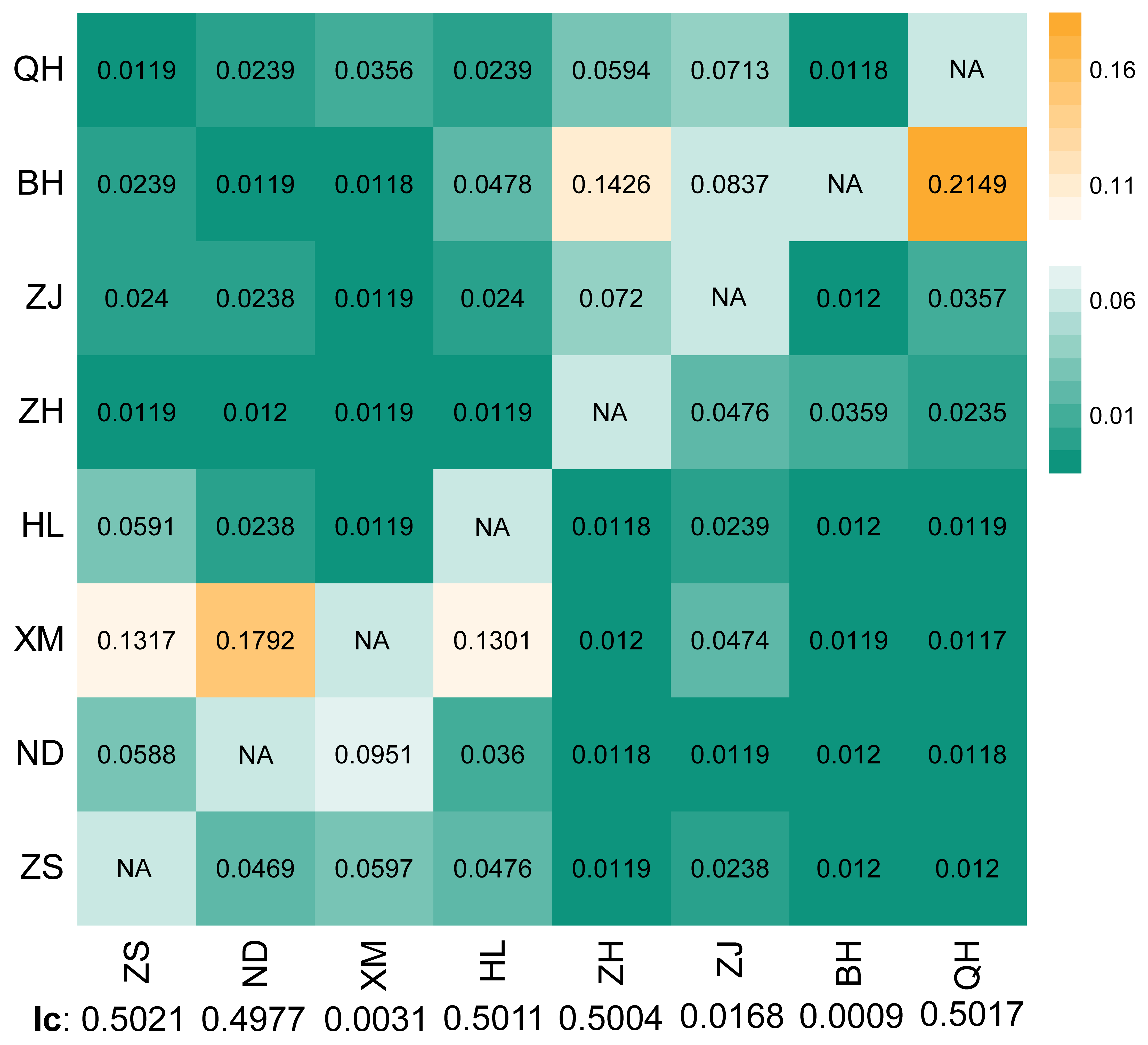

Supplement: Figure S1 — Venn diagram of SNP markers, between populations (A), HL and ZJ (B), and gender (C). [file DataSheet_1.zip › Figure S3.tif]

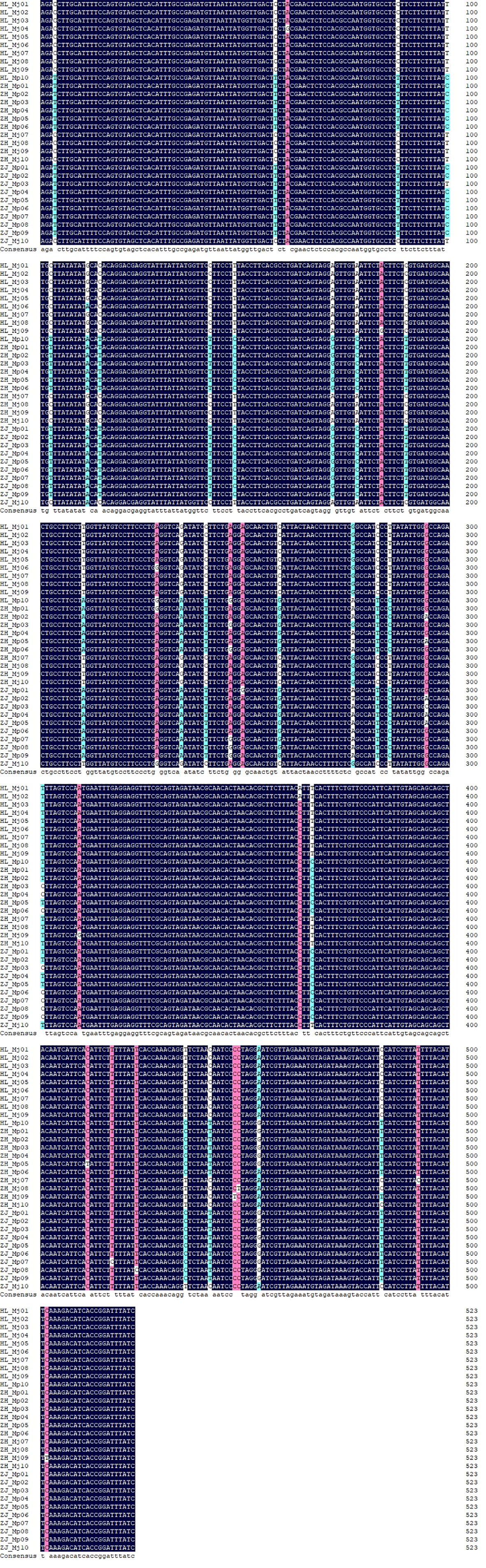

Supplement: Figure S1 — Venn diagram of SNP markers, between populations (A), HL and ZJ (B), and gender (C). [file DataSheet_1.zip › Figure S4.tif]

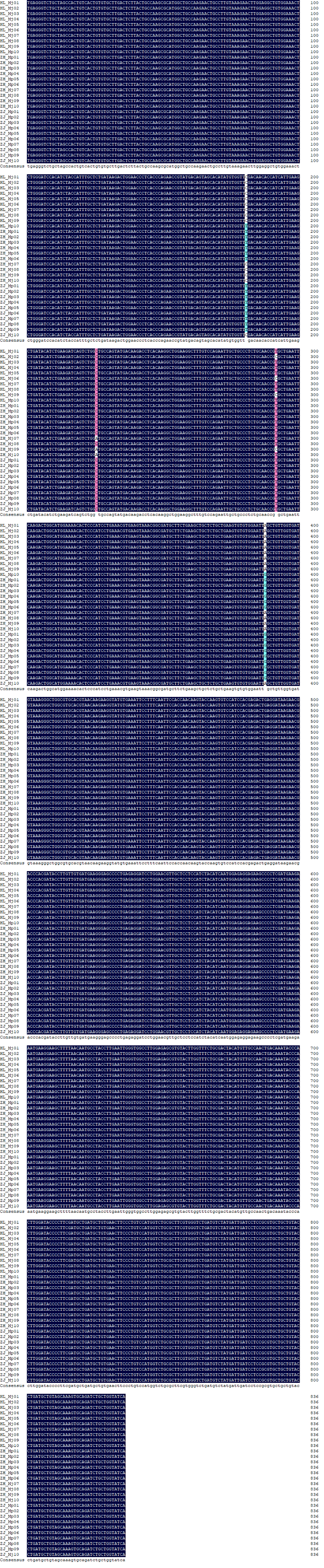

Supplement: Figure S1 — Venn diagram of SNP markers, between populations (A), HL and ZJ (B), and gender (C). [file DataSheet_1.zip › Figure S5.tif]
